# Improvement of PLLA Ductility by Blending with PVDF: Localization of Compatibilizers at Interface and Its Glycidyl Methacrylate Content Dependency

**DOI:** 10.3390/polym12081846

**Published:** 2020-08-17

**Authors:** Yan Zhang, Xiaoying Gu, Chunjun Ni, Fei Li, Yongjin Li, Jichun You

**Affiliations:** College of Materials, Chemistry and Chemical Engineering, Hangzhou Normal University, No. 2318 Yuhangtang Rd., Hangzhou 311121, China; ZhangyanHZNU@hotmail.com (Y.Z.); 13750803291@163.com (X.G.); ni_chunjun@163.com (C.N.); lifeiamazing@163.com (F.L.); yongjin-li@hznu.edu.cn (Y.L.)

**Keywords:** PLLA, ductility, reactive compatibilization, PVDF, interface

## Abstract

In this work, the localization of reactive compatibilizer (RC, containing poly(methyl methacrylate) (PMMA) backbone with randomly distributed glycidyl methacrylate (GMA) on it) at the polyvinylidene fluoride/poly(l-lactic acid) (PVDF/PLLA) interface has been manipulated by means of GMA contents. At the very beginning of mixing, RC tends to stay in the PVDF phase due to the miscibility between PVDF and PMMA. Upon further shearing, more and more PLLA chains have been grafted on PMMA backbone, producing PLLA–*g*–PMMA copolymer. The balanced stress on two sides accounts for the localization of compatibilizers at the PVDF/PLLA interface. Finally, the stress of the PLLA side has been enhanced remarkably due to the higher graft density of PLLA, resulting in the enrichment of the copolymer in the PLLA matrix. The migration of RC from the PVDF phase to the immiscible interface and PLLA matrix can be accelerated by employing RC with higher GMA content. Furthermore, the compatibilizer localization produces a significant influence on the morphology and ductility of the PVDF/PLLA blend. Only when the compatibilizers precisely localize at the interface, the blend exhibits the smallest domain and highest elongation at break. Our results are of great significance for not only the fabrication of PLLA with high ductility, but also the precise localization of compatibilizers at the interface of the immiscible blend.

## 1. Introduction

Poly(l-lactic acid) (PLLA) has been widely used in many fields due to its excellent biodegradability and biocompatibility [1,2]. The poor ductility of it, however, does hinder its applications [3]. Various strategies have been developed to prepare ductile PLLA. As a typical example, it has been blended with flexible polymers [4,5,6,7,8,9,10,11,12,13]. In these multicomponent blend systems, phase separation always occurs due to the poor miscibility of them [14,15]. Both physical and chemical compatibilizations have been employed to improve the compatibility of them [16,17]. In the former, it is hard for the premade compatibilizers to locate at the interface of immiscible polymer blend because of the “pull-in” and “pull-out” effects [18,19]. In the latter, the compatibilizers are obtained in situ at the interface [20,21,22,23,24,25]. During this process, however, they can migrate to the matrix or disperse phase upon further mixing and shearing. The migration and localization of them, dominated by mixing time, shear rate, diffusion behaviors and reactive group content, are key issues for fabricating ductile PLLA by blending with flexible polymer [26,27,28,29,30,31].

There have been some reports concerning the compatibilizer migration in the compatibilization in the polymer blend system with symmetrical composition. For instance, Jermi et al. investigated the reactive compatibilization by grafting of maleic anhydride (MAH) on polyamide 6 (PA6) with the help of benzoyl peroxide (BPO) [32]. The localization of compatibilizers can be adjusted by the concentration of BPO and MAH. In Monte Carlo simulation results, the compatibility of graft copolymer in homopolymer blends has been discussed in detail [33]. It has been revealed that the graft structures of various copolymers produced a significant effect on the compatibilization. The compatibilizers tended to localize at the interface of the immiscible blend and anchor two components in the case of longer side chains. The simulation conclusions were then validated in the work from Dong and his coworkers [8]. They successfully synthesized a series of reactive comb-like compatibilizers with different molecular architectures. Their results indicated that the localization of RC exhibited obvious dependence on the length of side chains. Li et al. prepared copolymers of methyl methacrylate–*co*–glycidyl methacrylate (MMA–*co*–GMA) which were used as the compatibilizers in the blend of PA6 and polyvinylidene fluoride (PVDF) [27]. They found that the GMA content produced a remarkable influence on the enrichment of compatibilizers. The best mechanical performance can be obtained in the specimen with 5% GMA.

**Scheme 1:**
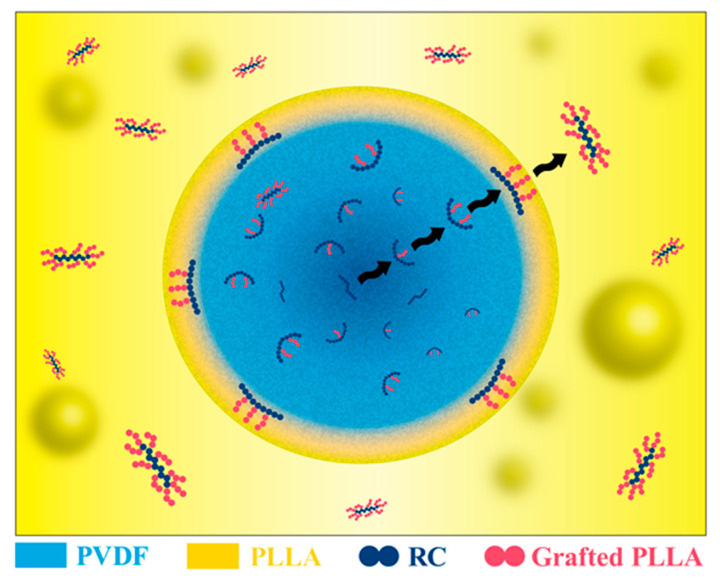
Schematic diagram of reactive compatibilizer migration and micelle formation.

In our previous work, a facile strategy has been developed to fabricate PLLA with excellent ductility by blending with a tiny amount of PVDF and reactive compatibilizers (RC) containing poly(methyl methacrylate) (PMMA) backbone with randomly distributed glycidyl methacrylate (GMA) [13]. The reaction between the terminal carboxyl group in PLLA and the epoxy groups in RC took place, producing PLLA–*g*–PMMA [8,20]. The resultant copolymer enhanced the compatibility between PVDF and PLLA, accounting for PLLA with high ductility. Obviously, the migration of compatibilizers, playing a key role in determining the phase-separated structures and mechanical performances, is sensitive to mixing time and GMA content. Inspired by the strategy for tailoring the migration of compatibilizers developed in polymer blend with symmetrical composition, it is proposed to manipulate the molecular architectures of PLLA–*g*–PMMA, the localization of the copolymer, and the resultant ductility of the PLLA/PVDF blend by employing compatibilizers with different GMA contents in this work (Scheme 1). When RC is added in the PVDF/PLLA blend, they tend to stay in PVDF phases due to the thermodynamic miscibility between PVDF and PMMA main chains. Then, more and more PLLA chains have been grafted on RC upon further mixing and shearing, resulting in the migration of them to the PVDF/PLLA interface or even PLLA matrix. In the case of lower GMA content, only a few PLLA chains can be grafted on compatibilizers, leading to the slower migration to the PVDF/PLLA interface. Higher GMA content accounts for the higher PLLA fraction in the copolymer of PLLA–*g*–PMMA, which is the reason for the enhanced stress to the PLLA matrix and the accelerated migration. According to this strategy, it is possible to prepare PLLA with high ductility based on the precise localization of compatibilizers at the PVDF/PLLA interface (Scheme 1).

## 2. Experimental Section

### 2.1. Materials

PVDF (KF850, *M*_w_ = 209,000, PDI = 2.0) was purchased from Kureha Chemicals (Tokyo, Japan) and PLLA (3001 D, *M*_w_ = 89,300, PDI = 1.8) was purchased from Nature works (Blair, NE, USA). The reactive compatibilizers (RC) with PMMA backbone and randomly distributed GMA were provided by Eco New Material Co., Ltd. (Hangzhou, China). Three kinds of RC named MG01, MG02 and MG03 represent the GMA feed ratio of 10, 20, and 30 wt % during synthesis. The Fourier Transform Infrared Spectrometer (FTIR, Bruker Optics, Ettlingen, Germany) results of them are shown in Figure S1 in the Supplementary Materials, in which the characteristic peaks of the epoxy group (at 909 cm^−1^) exhibit different transmittances.

### 2.2. Preparation of Polymer Blends

PLLA, PVDF and RC were dried in a vacuum oven at 80 °C overnight in order to remove moisture before melt blending. The weight ratio of PVDF/PLLA/RC was fixed at 5/95/3. The blends were prepared by melt blending in an internal mixer (Haake Polylab QC), (Thermo Fisher Scientific, Waltham, MA, USA) at a rotation speed of 20 rpm for 2 min and 50 rpm for the indicated time at 190 °C. After blending, all the blends were compression molded into film (thickness is 0.5 mm) under 10 MPa at 200 °C.

### 2.3. Tensile Property Tests

Tensile tests were performed by using a universal tensile testing machine (Instron, Norwood, MA, USA) at room temperature. The fixed tensile speed was 10 mm/min. The samples were cut into dumbbell shapes (18 mm × 0.5 mm × 3 mm) according to the ASTM D 412−80 standard. Each sample was tested for at least five times and the resulting values were averaged.

### 2.4. Dynamic Mechanical Analysis (DMA)

DMA was measured on the DMA Q800 (TA Instrument, New Castle, PA, USA) in nitrogen atmosphere. All specimens (8 mm × 6.30 mm × 0.50 mm) were heated from −80 to 200 °C with a heating rate of 3 °C/min. A tensile mode with 10 µm of amplitude and 5 Hz of frequency was applied.

### 2.5. Microstructure Characterization

The sea-island structure of samples was observed by scanning electron microscopy (Hitachi S-4800, Tokyo, Japan) with an accelerating voltage of 3.0 kV. The specimens were fractured in liquid nitrogen and sputter-coated with gold before observation. The migration process and micelle formation were observed by transmission electron microscopy (Hitachi HT-7700, Tokyo, Japan) with an accelerating voltage of 60.0 kV. Each specimen was sliced into 80 nm sections by microtome. PVDF was stained by RuO_4_ (Ruthenium tetroxide) for 4 h to provide better contrast under TEM.

### 2.6. Rheology Characterization

Rheology measurements were carried out on the MCR301 rheometer (Anton Paar Instrument, Graz, Austria) at 200 °C. A parallel plate configuration was used with a gap size of 1 mm and a plate diameter of 25 mm. The strain was 0.05, and the sweep frequency ranged from 0.01 to 100 rad/s.

### 2.7. Particle Size of Statistics

Particle size statistics were carried out by Nano Measurer (Department of Chemitry, Fudan University, Shanghai, China, software version: 1.2.5). Relevant data were obtained through the particle size statistics and analysis of PVDF phases in SEM images.

## 3. Results and Discussions

**Figure 1:**
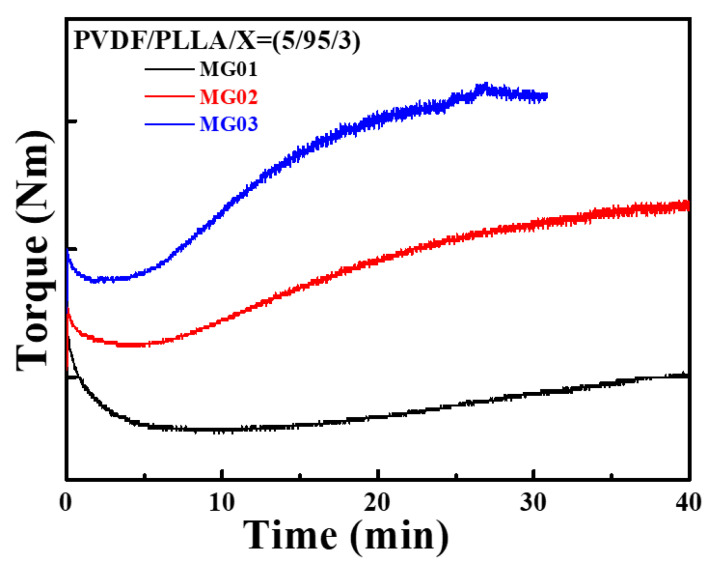
Time–torque curves of the polyvinylidene fluoride/poly(l-lactic acid) (PVDF/PLLA) blends with various compatibilizers and mixing time.

According to our previous work, PVDF/PLLA/RC blend with a weight ratio of 5/95/3 exhibits excellent ductility [13]. In this work, therefore, the same weight ratio has been adopted. Three reactive compatibilizers with different GMA contents (MG01, MG02 and MG03 representing the GMA feed ratio of 10, 20, and 30 wt % during synthesis respectively, Figure S1) have been employed. The time–torque curves of the PVDF/PLLA blends with reactive compatibilizers containing different GMA contents were shown in Figure 1. The torque rises rapidly and then decreases, which can be attributed to the addition and melting of materials respectively. After that, the torque increases because of the chemical reactions between the carboxyl group in PLLA and the epoxy groups in RC. This reaction has been discussed in detail and the reaction equations can be found in our previous works [20,29,34]. Obviously, the occurrence of a torque plateau means that the graft of reactive compatibilizer on the PMMA backbone has finished [35]. In the black curve (MG01), there is no plateau even when the blend has been mixed for 40 min. It takes ~35 min to reach the plateau in the specimen of MG02 (red curve). In the case of MG03 (blue curve), however, the time has been shortened to ~20 min. In the following sections, therefore, four specimens have been prepared upon mixing for different periods to investigate the migration and localization of RC as well as its effect on morphology and mechanical performance.

**Figure 2:**
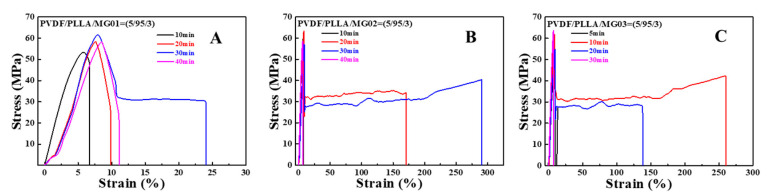
Stress–strain curves of the PVDF/PLLA blends with various compatibilizers and mixing time: MG01 (**A**), MG02 (**B**), MG03 (**C**).

As shown in Figure S2 in the Supplementary Materials, neat PLLA exhibits high yielding strength (~60 MPa) but low elongation at break (~10%). Upon blending with PVDF, neither the strength nor the elongation at break increased due to the poor interaction between PVDF and PLLA [13]. Figure 2 shows the stress–strain curves of the PVDF/PLLA blends with different compatibilizers upon mixing for the indicated time. The corresponding results are summarized in Table 1. When MG01 is adopted (Figure 2A), all specimens are brittle. In the case of MG02, the PVDF/PLLA blend has the maximum elongation at break at the mixing time of 30 min, reaching 291% in Figure 2B. The blend of PVDF/PLLA/MG03 exhibits the elongation at break of 273% when it is mixed for 10 min (Figure 2C). The comparison of mechanical properties among these specimens indicates that there is an optimal mixing time for the improvement of ductility, at which the elongation at break of the blend system reaches the maximum magnitude. Furthermore, the optimal mixing time exhibits crucial dependence on the GMA content of reactive compatibilizer. It decreases upon increasing the GMA content in reactive compatibilizers.

**Figure 3:**
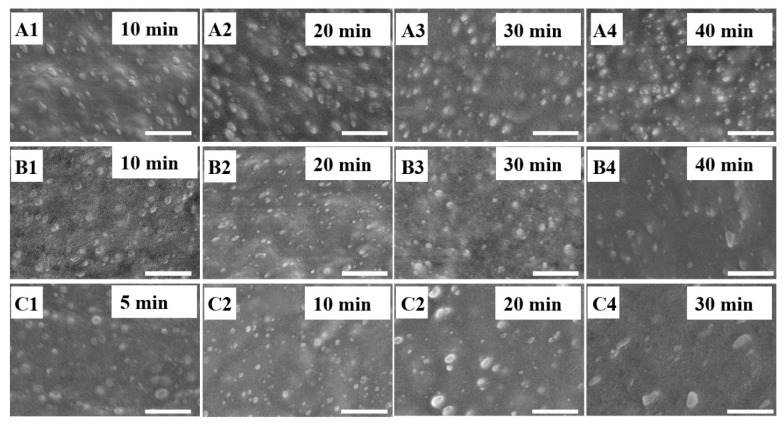
SEM images of PVDF/PLLA/RC of MG01 (**A1**–**A4**), MG02 (**B1**–**B4**), MG03 (**C1**–**C4**) mixed for the indicated time. The scale bar is 1 micron.

**Figure 4:**
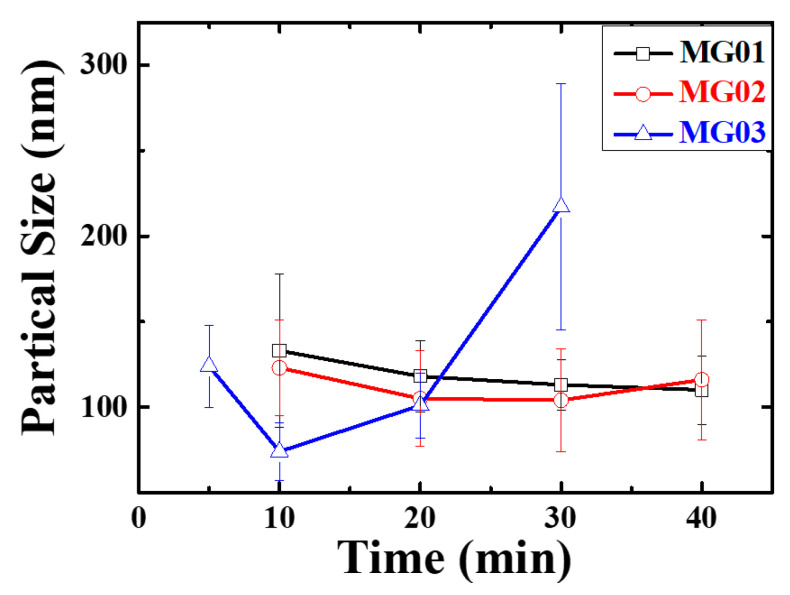
PVDF phase domain size as a function of mixing time and compatibilizers.

SEM has been utilized to investigate the morphology evolution of the PVDF/PLLA blends with different compatibilizers (Figure 3). In all specimens, there are sea-island structures. By means of volume fraction calculation, it can be validated that the matrix and dispersed phases are PLLA and PVDF respectively. As shown in many reports, PVDF/PLLA is a typical immiscible blend system, exhibiting big domains and poor interface [8,15,36,37]. In this work, however, the interfacial adhesion between them has been enhanced remarkably due to the existence of reactive compatibilizers, which can be seen from the emulsified interface. In Figure 3(A1–A4), the PVDF islands exhibit a diameter of 133 nm (at 10 min, Figure 3(A1)). This value is much lower than that in the PVDF/PLLA blend without compatibilizers (~1 micron in ref [13]). Upon further mixing, the structure and its size do not change obviously, which has been shown in the particle size statistics (Table S1 in the Supplementary Materials and Figure 4). In the case of MG02 (Figure 3(B1–B4)), the PVDF domain size decreases from 123 (Figure 3(B1)) to 105 and 104 nm (Figure 3(B2,B3)). Then, it increases to 116 nm (Figure 3(B4)). Furthermore, the interface has been enhanced from Figure 3(B1–B3). The same thing happens in Figure 3(C1–C4). The size of PVDF islands first decreases (from 124 to 74 nm, Figure 3(C1,C2)) and then increases (to 217 nm, Figure 3(C4)). According to the discussion above, it is the PVDF domain size (Figure 4) and PVDF/PLLA interface that determine the ductility of the PVDF/PLLA blend (Figure 2 and Table 1). The smaller domain and emulsified interface correspond to the higher ductility. Then, what is the reason for the morphology evolution shown in Figure 3? Added in the PVDF/PLLA blend, the reactive compatibilizers (RC) tend to mix with the PVDF phase because of the excellent miscibility between PVDF and PMMA. During mixing, the reaction between the terminal carboxyl group in PLLA and the epoxy groups in RC takes place, which is the reason for the increase of torque shown in Figure 1. Then, more and more PLLA chains have been grafted on PMMA backbone, producing PLLA–*g*–PMMA copolymer. As a result, the stress from the PLLA side in the PVDF/PLLA blend is enhanced significantly, accounting for the migration of RC from PVDF to interface and even PLLA matrix. Due to the low GMA content, the migration of grafted MG01 has not finished within 40 min (black curve in Figure 1), so PVDF domains do not change significantly (Figure 3(A1–A4) and Figure 4). However, MG02 and MG03 with high GMA contents accelerate the migration remarkably. During this process, the compatibilizer concentration at the interface is the highest at a certain moment (20 min for MG02 and 10 min for MG03), which is the reason for the smaller PVDF domain, the emulsified PVDF/PLLA interface and higher ductility. In other words, the migration of the compatibilizer from the PVDF phase to the interface plays a key role in the improvement of the PVDF/PLLA ductility. Upon further mixing, PVDF particle size increases since the compatibilizer migrates from the interface to the PLLA matrix. Consequently, the elongation at break of the blend exhibits lower magnitudes (Figure 2 and Table 1) [28,29,30,38,39,40].

**Figure 5:**
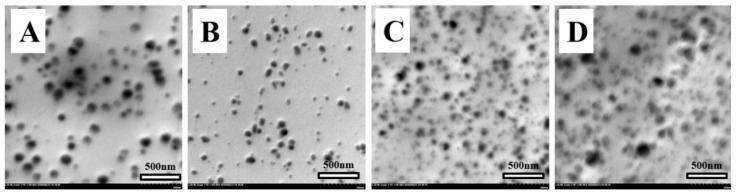
TEM images of PVDF/PLLA/MG02(5/95/3) upon mixing for 10 min (**A**), 20 min (**B**), 30 min (**C**) and 40 min (**D**).

To validate the migration of compatibilizers, TEM measurements have been performed by taking PVDF/PLLA/MG02 (5/95/3) as an example. In Figure 5, black and white parts correspond to PVDF and PLLA, respectively. When the mixing time is 10 min, there are PVDF domains with a diameter of ~120 nm (Figure 5A). Upon further mixing, our attention should be paid to the following issues. On one hand, the PVDF domain size decreases from ~120 to ~100 nm (Figure 5B,C). At last, it increases to ~200 nm (Figure 5D); on the other hand, there are no gray domains in Figure 5A,B while the gray micelles are obvious in Figure 5C,D. The evolution of PVDF domain size and the formation of micelles in the PLLA matrix can act as the direct evidence for the migration of compatibilizers from PVDF to interface and PLLA matrix.

**Figure 6:**
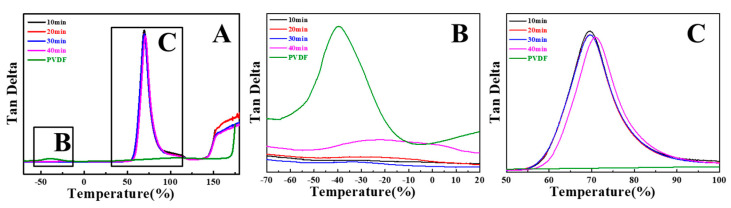
PVDF/PLLA/MG02 blends with various mixing time: complete dynamic mechanical analysis (DMA) curves (**A**), PVDF glass transition temperature region (**B**) and PVDF glass transition temperature region (**C**).

DMA has been used to investigate the relaxation of the PLLA matrix as well as the interface in the PVDF/PLLA blends (Figure 6 and Table 2) by taking PVDF/PLLA/MG02 as an example. In Figure 6A, we can find only a relaxation at ~70 °C. To show other relaxation clearly, our attention has been paid to Figure 6B in the indicated temperature range. In the result of neat PVDF, there is a glass transition at about −40 °C. In the black and red curves, however, we cannot find this transition. When the specimen is mixed for 30 min (blue curve), there is a broad peak. It becomes more and more obvious upon further mixing (pink curve) for 40 min. This evolution indicates that the glass transition of PVDF has been affected by PMMA in RC at the blending time of 20 and 30 min. Finally (40 min), the influence becomes neglectable. Figure 6C shows the glass transition of PLLA. The glass transition temperature of it decreases slightly (from 69.7 to 69.6 °C) and then increases to 70.7 °C when it is mixed for 40 min (Figure 6C and Table 2). In addition, the intensity and the half-peak-width of PLLA glass transition can be used to describe its relaxation [13]. The former increases while the latter decreases from 30 to 40 min, suggesting that PLLA exhibits different relaxation behaviors relative to neat PLLA. The evolution of glass transition temperature, the intensity and half-peak-width can be attributed to the influence of grafted PLLA. This result makes it clear that reactive compatibilizers locate in the PLLA matrix upon mixing for 40 min.

According to SEM, TEM and DMA results, we can describe the migration of compatibilizers and the resultant mechanical performance as follows. When the compatibilizers are blended with PVDF and PLLA, they tend to stay in the PVDF phase because of the excellent miscibility between them [8,15,20,41,42,43,44]. This is the reason for the disappearance of the PVDF glass transition peak in DMA curves (black curve in Figure 6B) and the absence of micelles in Figure 5A. In this case, PVDF and PLLA have not been compatibilized well, leading to the bigger domains (Figure 3(A1,B1,C1)) and poor mechanical performance (black curves in Figure 2). Upon mixing and shearing, the compatibilizers can contact PLLA, resulting in the reaction between the terminal carboxyl group (in PLLA) and the epoxy groups (in RC). In the subsequently grafted copolymer of PLLA–*g*–PMMA, therefore, there are two kinds of entanglement including PMMA with PVDF and grafted PLLA with free PLLA. The balanced stress in two sides accounts for the precise localization of the compatibilizers at the interface of PVDF/PLLA, corresponding to the migration of RC from PVDF islands to interface. This is the reason for the smallest domain size (Figure 3(B3,C2)) and the highest elongation at break (blue curve in Figure 2B and red curve in Figure 2C). The glass transition of PVDF becomes clear (blue curve in Figure 6B) relative to the result of 10 min. At the same time, the grafted PLLA produces a certain influence on the relaxation of free PLLA (Figure 6C). With further increase of mixing time, more and more PLLA chains have been grafted on the compatibilizers, producing asymmetric stress in the copolymer. As a result of enhanced entanglement of grafted PLLA with free PLLA, the compatibilizers migrate from PVDF/PLLA interface to the PLLA matrix. The glass transition temperature of PLLA increases since it exhibits certain miscibility with PMMA (Figure 6C). On the contrary, the glass transition of PVDF becomes obvious in Figure 6B (pink curve) because of the localization of RC in the PLLA matrix. In this case, the compatibilization becomes less efficient, resulting in the occurrence of bigger PVDF domains (Figure 3, Figure 4 and Figure 5), micelles in the PLLA matrix (Figure 5D) and lower magnitudes of elongation at break (pink curves in Figure 2). In other words, the compatibilizers migrate from PVDF to the PVDF/PLLA interface, enhancing the compatibilization effect (10–20 min). Then, they move to the PLLA matrix because of the higher graft density of PLLA, accounting for the poor compatibilization effect (30–40 min).

**Figure 7:**
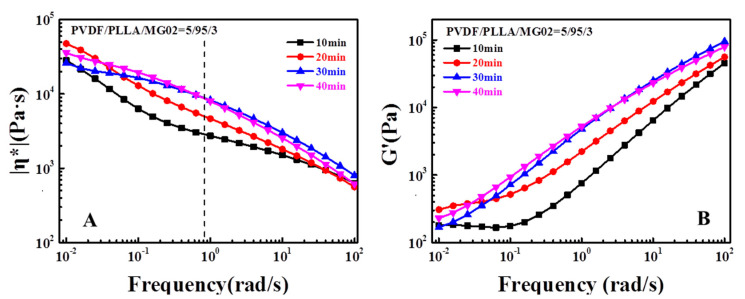
Complex viscosity (**A**), storage modulus (**B**) as a function of frequency at 200 °C for PVDF/PLLA various compatibilizers and mixing time.

The evolution discussed above can be validated by means of rheology measurement. Figure 7A displays the complex viscosity (η*) of PVDF/PLLA/MG02 blends with different mixing times as a function of frequencies. All the blends show shear thinning behavior. The viscosities at the indicated angular frequency (dash line: 50 rpm corresponds to 0.83 rad/s) increase and then decrease. The viscosity shows the highest magnitude when the blending time is 30 min. The compatibilization effect can be assessed by means of frequency dependence of storage modulus as shown in Figure 7B. In the result of mixing for 10 min (black curve), there is a slight shoulder in the low-frequency region, which can be attributed to the poor compatibilization effect due to the localization of RC in the PVDF phase [45]. Upon further mixing, this shoulder disappears, producing the straight line (blue curve). In this case, it is the precise localization of PLLA–*g*–PMMA copolymer at the PVDF/PLLA interface that enhances the compatibilization. Finally, the storage modulus deviates from the straight line again in the pink curve in the low-frequency region, corresponding to the migration of RC from the interface to the PLLA matrix (Figure 5D).

## 4. Conclusions

In this work, reactive compatibilizers with different GMA contents have been employed to fabricate ductile PLLA by blending with a tiny amount of PVDF. The reaction between the epoxy group in RC and the terminal carboxyl group in PLLA produces the copolymer of PLLA–*g*–PMMA. This copolymer can act as compatibilizers since PMMA backbone and grafted PLLA can entangle with PVDF and free PLLA respectively. At the very beginning of mixing, the reactive compatibilizers tend to stay in PVDF domains due to the thermodynamic miscibility between PVDF and PMMA. Upon further mixing, some PLLA chains have been grafted on RC. The copolymer migrates to the PVDF/PLLA interface because of the stress balance on two sides. Finally, there are so many PLLA chains grafted on RC that the compatibilizers are pulled into the PLLA matrix. In the case of lower GMA content, there are only a few PLLA chains grafted on RC, leading to the slow migration. When the GMA content is high, the enhanced stress in the PLLA side accelerates the migration. Therefore, the precise localization of compatibilizers at the PVDF/PLLA interface and the fabrication of PLLA with high ductility have been achieved successfully by tailoring GMA content. Our results are of great significance for not only the fabrication of PLLA with high ductility, but also the precise localization of compatibilizers at the interface of the immiscible blend. 

## Figures and Tables

**Table 1:** Mechanical properties of the PVDF/PLLA blends with various compatibilizers and mixing time.

Sample	Mixing Time (min)	E (GPa)	σ_y_ (MPa)	σ_b_ (MPa)	ε (%)
PVDF\PLLA\MG01 (5\95\3)	10 min	1.0 ± 0.1	51.1 ± 2.6	46.7 ± 3.9	8 ± 0
	20 min	1.2 ± 0.1	60.2 ± 0.1	45.4 ± 12.9	13 ± 3
	30 min	1.1 ± 0.3	62.3 ± 0.8	42.1 ± 11.3	19 ± 6
	40 min	1.3 ± 0.1	61.5 ± 0.2	46.9 ± 13.2	14 ± 3
PVDF/PLLA/MG02 (5/95/3)	10 min	1.3 ± 0.0	58.6 ± 0.0	50.8 ± 9.2	8 ± 2
	20 min	1.1 ± 0.0	60.1 ± 3.2	32.9 ± 0.7	143 ± 57
	30 min	1.2 ± 0.0	56.5 ± 0.6	39.1 ± 2.0	291 ± 29
	40 min	1.3 ± 0.1	59.8 ± 0.0	52.5 ± 3.0	14 ± 10
PVDF/PLLA/MG03 (5/95/3)	5 min	1.1 ± 0.1	/	54.9 ± 1.6	10 ± 0
	10 min	1.0 ± 0.1	60.5 ± 1.4	42.8 ± 1.6	273 ± 25
	20 min	1.2 ± 0.1	57.3 ± 1.2	31.6 ± 2.2	138 ± 44
	30 min	1.2 ± 0.0	/	62.9 ± 0.8	7.7 ± 0

“/”: sample break directly when stretched, so there’s no yield strength.

**Table 2:** Comparison table of DMA data and elongation at break.

Sample	Mixing Time (min)	ε (%)	*T*g (°C)	Half Peak Width
PVDF/PLLA/MG02 (5/95/3)	10	7.9 ± 1.5	69.7	11.3
	20	142.7 ± 57.0	69.6	11.6
	30	290.7 ± 28.5	69.6	11.6
	40	14.1 ± 10.3	70.7	11.3

## Supplementary Materials

The following are available online at https://www.mdpi.com/2073-4360/12/8/1846/s1, Figure S1. FTIR of three reactive compatibilizers. The curves were normalized according to peak at 2950 cm^−1^. Three kinds of RC named as MG01, MG02 and MG03 represent the GMA feed ratio of 10, 20 and 30 wt % during synthesis. The transmittances of epoxy group at 909 cm^−1^ exhibit lower magnitudes (from MG01, MG02 to MG03), indicating the highest GMA content in MG03. Figure S2. Strain-stress curves of neat PLLA and PLLA/PVDF blend (5/95, without compatibilizers). Table S1. Particle size statistics of PVDF/PLLA blends with different compatibilizers and mixing times.

## References

[1] Hamad K., Kaseem M., Ayyoob M., Joo J., Deri F. (2018). Polylactic acid blends: The future of green, light and tough. Prog. Polym. Sci..

[2] Zhu Y.Q., Romain C., Williams C.K. (2016). Sustainable polymers from renewable resources. Nature.

[3] Nofar M., Sacligil D., Carreau P.J., Kamal M.R., Heuzey M.C. (2019). Poly (lactic acid) blends: Processing, properties and applications. Int. J. Biol. Macromol..

[4] Chen Y., Pan M.W., Li Y., Xu J.Z., Zhong G.J., Ji X., Yan Z., Li Z.M. (2018). Core-shell nanoparticles toughened polylactide with excellent transparency and stiffness-toughness balance. Compos. Sci. Technol..

[5] Deng S.H., Bai H.W., Liu Z.W., Zhang Q., Fu Q. (2019). Toward supertough and heat-resistant stereocomplex-type polylactide/elastomer blends with impressive melt stability via in situ formation of graft copolymer during one-pot reactive melt blending. Macromolecules.

[6] Dong W.Y., He M.F., Wang H.T., Ren F.L., Zhang J.Q., Zhao X.W., Li Y.J. (2015). PLLA/ABS blends compatibilized by reactive comb polymers: Double *T*_g_ depression and significantly improved toughness. ACS Sustain. Chem. Eng..

[7] Dong W.Y., Jiang F.H., Zhao L.P., You J.C., Cao X.J., Li Y.J. (2012). PLLA microalloys versus PLLA nanoalloys: Preparation, morphologies, and properties. ACS Appl. Mater. Inter..

[8] Dong W.Y., Wang H.T., Ren F.L., Zhang J.Q., He M.F., Wu T., Li Y.J. (2016). Dramatic improvement in toughness of PLLA/PVDF blends: The effect of compatibilizer architectures. ACS Sustain. Chem. Eng..

[9] Oyama H.T. (2009). Super-tough poly(lactic acid) materials: Reactive blending with ethylene copolymer. Polymer.

[10] Wang Q.J., Zhang J., Wang X.H., Wang Z.G. (2020). Significant enhancement of notched Izod impact strength of PLA-based blends through encapsulating PA11 particles of low amounts by EGMA elastomer. Appl. Surf. Sci..

[11] Wu B.G., Zeng Q.T., Niu D.Y., Yang W.J., Dong W.F., Chen M.Q., Ma P.M. (2019). Design of supertoughened and heat-resistant PLLA/elastomer blends by controlling the distribution of stereocomplex crystallites and the morphology. Macromolecules.

[12] Yang X., Wang H.T., Chen J.L., Fu Z.A., Zhao X.W., Li Y.J. (2019). Copolymers containing two types of reactive groups: New compatibilizer for immiscible PLLA/PA11 polymer blends. Polymer.

[13] Zhang Y., Li F., Yu Q.L., Ni C.J., Gu X.Y., Li Y.J., You J.C. (2019). Fabrication of PLLA with high ductility and transparence by blending with tiny amount of PVDF and compatibilizers. Macromol. Mater. Eng..

[14] Wang H.T., Fu Z.A., Dong W.Y., Li Y.J., Li J.Y. (2016). Formation of interfacial janus nanomicelles by reactive blending and their compatibilization effects on immiscible polymer blends. J. Phys. Chem. B.

[15] Li F., Zhao X.W., Wang H.T., Chen Q., Wang S.H., Chen Z.H., Zhou X.Y., Fan W.C., Li Y.J., You J.C. (2019). Sub-100 nm co-continuous structures fabricated in immiscible commodity polymer blend with extremely low volume/viscosity ratio. ACS Appl. Polym. Mater..

[16] Jeon H.K., Zhang J., Macosko C.W. (2005). Premade vs. reactively formed compatibilizers for PMMA/PS melt blends. Polymer.

[17] Koulic C., Yin Z., Pagnoulle C., Gilbert B., Jérôme R. (2001). Premade versus in situ formed compatibilizer at the PS/PMMA interface: Contribution of the Raman confocal microscopy to the fracture analysis. Polymer.

[18] Macosko C., Guégan P., Khandpur A. (1996). Compatibilizers for melt blending:  Premade block copolymers. Macromolecules.

[19] Filippi S., Yordanov H., Minkova L., Polacco G., Talarico M. (2004). Reactive compatibilizer precursors for LDPE/PA6 blends, 4: Maleic anhydride and glycidyl methacrylate grafted SEBS. Macromol. Mater. Eng..

[20] Dong W.Y., Wang H.T., He M.F., Ren F.L., Wu T., Zheng Q.R., Li Y.J. (2015). Synthesis of reactive comb polymers and their applications as a highly efficient compatibilizer in immiscible polymer blends. Ind. Eng. Chem. Res..

[21] Fang H.G., Jiang F., Wu Q.H., Ding Y.S., Wang Z.G. (2014). Supertough polylactide materials prepared through in situ reactive blending with PEG-based diacrylate monomer. ACS Appl. Mater. Inter..

[22] Fu Z.A., Wang H.T., Zhao X.W., Horiuchi S., Li Y.J. (2017). Immiscible polymer blends compatibilized with reactive hybrid nanoparticles: Morphologies and properties. Polymer.

[23] Kim H.Y., Ryu D.Y., Jeong U., Kho D.H., Kim J.K. (2005). The effect of chain architecture of in situ formed copolymers on interfacial morphology of reactive polymer blends. Macromol. Rapid. Commun..

[24] Kim J.K., Lee H. (1996). The effect of PS—GMA as an in situ compatibilizer on the morphology and rheological properties of the immiscible PBT/PS blend. Polymer.

[25] Wei B., Lin Q.Q., Zheng X., Gu X.Y., Zhao L., Li J.C., Li Y.J. (2019). Reactive splicing compatibilization of immiscible polymer blends: Compatibilizer synthesis in the melt state and compatibilizer architecture effects. Polymer.

[26] Dong W.Y., Hakukawa H., Yamahira N., Li Y.J., Horiuchi S. (2019). Mechanism of reactive compatibilization of PLLA/PVDF blends investigated by scanning transmission electron microscopy with energy-dispersive X-ray spectrometry and electron energy loss spectroscopy. ACS Appl. Polym. Mater..

[27] Li D.F., Song S.X., Li C., Cao C.L., Sun S.L., Zhang H.X. (2015). Compatibilization effect of MMA–*co*–GMA copolymers on the properties of polyamide 6/poly(vinylidene fluoride) blends. J. Polym. Res..

[28] Mechbal N., Bousmina M. (2007). Effect of copolymer addition on drop deformation during uniaxial elongation and during relaxation after cessation of flow. Macromolecules.

[29] Scott C.E., Macosko C.W. (1994). Morphology development during reactive and non-reactive blending of an ethylene-propylene rubber with two thermoplastic matrices. Polymer.

[30] Sundararaj U., Macosko C., Rolando R., Chan H. (1992). Morphology development in polymer blends. Polym. Eng. Sci..

[31] Sundararaj U., Macosko C.W. (1995). Drop breakup and coalescence in polymer blends: The effects of concentration and compatibilization. Macromolecules.

[32] Jermi A., He Y.D., Khan Q., Wahab N. (2018). Barrier, mechanical, morphological and thermal properties of compatibilized high density polyethylene and polyamide 6 blends. Polym. Sci. Ser. B.

[33] Zhu Y.T., Ma Z.W., Li Y.Q., Cui J., Jiang W. (2007). Monte Carlo simulation of the compatibility of graft copolymer compatibilized two incompatible homopolymer blends: Effect of graft structure. J. Appl. Polym. Sci..

[34] Scott C.E., Macosko C.W. (1991). Model experiments concerning morphology development during the initial stages of polymer blending. Polym. Bull..

[35] Zhang Y., Song J.X., Jin X.H., Li F., Li Y.J., You J.C. (2020). Direct evidence for the validity of assessing reaction extent by torque spectrum during reactive processing. Polymer.

[36] Li F., Zhang Y., Zhao X.W., Chen Q., Li Y.J., You J.C. (2019). Graft ratio: Quantitative measurement and direct evidence for its blending sequence dependence during reactive compatibilization in PVDF/PLLA. Polymer.

[37] Pan P.J., Shan G.R., Bao Y.Z. (2014). Enhanced nucleation and crystallization of poly(l-lactic acid) by immiscible blending with poly(vinylidene fluoride). Ind. Eng. Chem. Res..

[38] Karger-Kocsis J., Kalló A., Kuleznev V.N. (1984). Phase structure of impact-modified polypropylene blends. Polymer.

[39] Shi T.F., Ziegler V.E., Welge I.C., An L.J., Wolf B.A. (2004). Evolution of the interfacial tension between polydisperse “immiscible” polymers in the absence and in the presence of a compatibilizer. Macromolecules.

[40] Walther A., Matussek K., Muller A.H.E. (2008). Engineering nanostructured polymer blends with controlled nanoparticle location using Janus particles. ACS Nano.

[41] Martínez-Salazar J., Canalda J.C., Vallejo B. (1994). Phase separation studies on poly(vinylidene fluoride) and poly(methyl methacrylate) quenched blends. Macromol. Symp..

[42] Canalda J.C., Hoffmann T., Martínez-Salazar J. (1995). On the melting behaviour of polymer single crystals in a mixture with a compatible polymer: 1. Poly(vinylidene fluoride)/poly(methyl methacrylate) blends. Polymer.

[43] Mijovic J., Sy J.W., Kwei T.K. (1997). Reorientational dynamics of dipoles in poly(vinylidene fluoride)/poly(methyl methacrylate) (PVDF/PMMA) blends by dielectric spectroscopy. Macromolecules.

[44] Chen D.P., Wang H.T., Li Y.J. (2017). Reactive compatibilization: Formation of double-grafted copolymers by in situ binary grafting and their compatibilization effect. ACS Appl. Mater. Interfaces.

[45] Li R.M., Yu W., Zhou C.X. (2007). Investigation of phase separation in a partially miscible polymer blend by rheology. J. Macromol. Sci. B.

