# Supplementary material for: Improvement of PLLA Ductility by Blending with PVDF: Localization of Compatibilizers at Interface and Its Glycidyl Methacrylate Content Dependency
Source: Polymers (Basel). 2020 Aug 17;12(8):1846. doi: 10.3390/polym12081846 (PMC7464155; doi:10.3390/polym12081846)
Supplement: Supplementary file 1 [file polymers-12-01846-s001.pdf]

Supplementary Materials

Improvement of PLLA Ductility by Blending with PVDF: Localization of Compatibilizers and its GMA Content Dependency

Yan Zhang, Xiaoying Gu, Chunjun Ni, Fei Li, Yongjin Li and Jichun You *

College of Materials, Chemistry and Chemical Engineering, Hangzhou Normal University, No. 2318Yuhangtang Rd., Hangzhou 311121, China; ZhangyanHZNU@hotmail.com (Y.Z.); 13750803291@163.com(X.G.); ni_chunjun@163.com(C.N.); lifeiamazing@163.com(F.L.); yongjin-li@hznu.edu.cn(Y.L.)

* Correspondence: author: you@hznu.edu.cn

Received: 13 July 2020; Accepted: 13 August 2020; Published: date

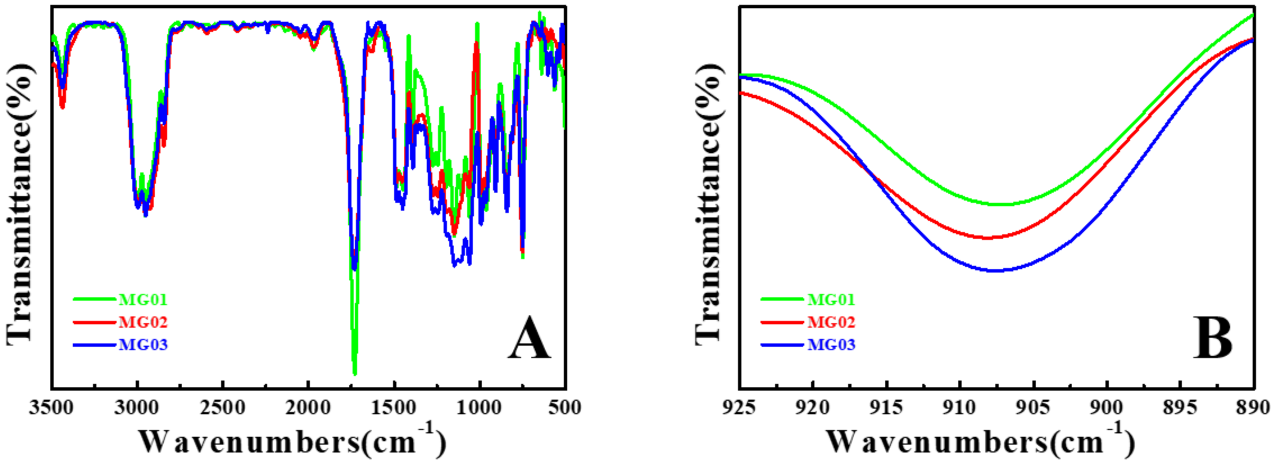

Figure S1. FTIR of three reactive compatibilizers. The curves were normalized according to peak at 2950cm^{-1}. Three kinds of RC named as MG01, MG02 and MG03 represent the GMA feed ratio of 10, 20 and 30 wt % during synthesis. The transmittances of epoxy group at 909cm^{-1} exhibit lower magnitudes (from MG01, MG02 to MG03), indicating the highest GMA content in MG03.

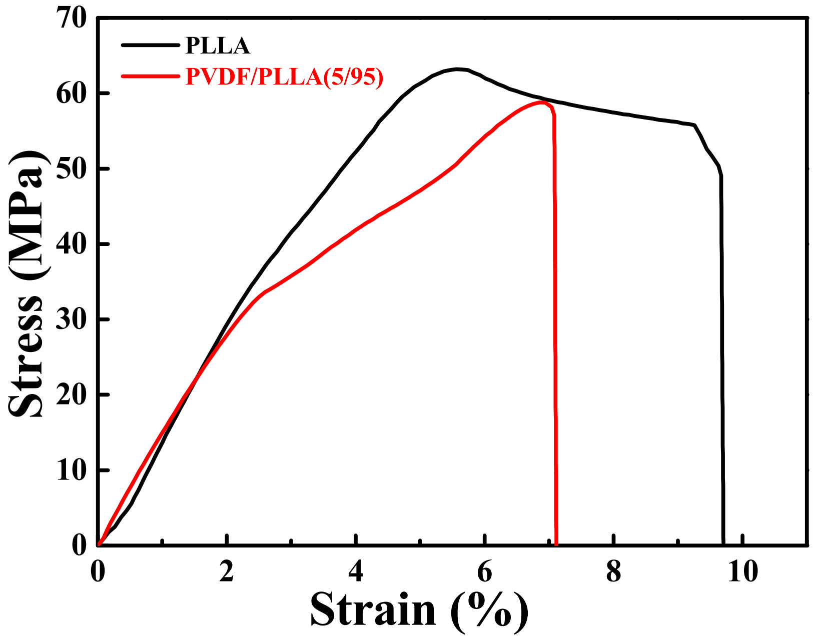

Figure S2. Strain-stress curves of neat PLLA and PLLA/PVDF blend (5/95, without compatibilizers).

Table S1. Particle size statistics of PVDF/PLLA blends with different compatibilizers and mixing times.

Sample	Mixing Time (min)	Partical Size (nm)
PVDF\PLLA\MG01 (5\95\3)	10	133 ± 45
	20	118 ± 21
	30	113 ± 15
	40	110 ± 20
PVDF/PLLA/MG02 (5/95/3)	10	123 ± 28
	20	105 ± 28
	30	104 ± 30
	40	116 ± 35
PVDF/PLLA/MG03 (5/95/3)	5	124 ± 24
	10	74 ± 17
	20	101 ± 19
	30	217 ± 72
